# The Role of Thyroid Hormones and Autoantibodies in Metabolic Dysfunction Associated Fatty Liver Disease: TgAb May Be a Potential Protective Factor

**DOI:** 10.3389/fendo.2020.598836

**Published:** 2020-12-08

**Authors:** Xiaofu Zhang, Ruyi Li, Yingjie Chen, Yuning Dai, Ling Chen, Lei Qin, Xingbo Cheng, Yan Lu

**Affiliations:** ^1^ Department of Clinical Medicine, Medical College of Soochow University, Suzhou, China; ^2^ Department of Preventive Medicine and Public Health, Medical College of Soochow University, Suzhou, China; ^3^ Department of Endocrinology, The First Affiliated Hospital of Soochow University, Suzhou, China; ^4^ Department of General Surgery, The First Affiliated Hospital of Soochow University, Suzhou, China

**Keywords:** metabolic dysfunction associated fatty liver disease, thyroid autoantibodies, thyroid hormones, high-sensitive C-reactive protein, inflammation

## Abstract

**Background:**

Previous studies have shown that metabolic dysfunction associated fatty liver disease (MAFLD) is associated with thyroid hormones (THs), immunity, and inflammation status, but few studies involved thyroid autoimmunity. This study aimed to evaluate the role of THs, thyroid autoantibodies, inflammatory biomarkers in MAFLD, its cofactors, and other possible determinants.

**Materials and Methods:**

In the study, a total of 424 Chinese patients were selected and categorized as non-MAFLD and MAFLD. Serum thyroid hormone, thyroid autoantibody and high-sensitive C-reactive protein (hsCRP) levels were measured. The data of blood pressure, the serum lipid profile, glucose and liver enzymes were collected. The differences and association between research findings were examined and analyzed by Wilcoxon Signed Rank Test, One-Way ANOVA test and Multiple Logistic Regression models.

**Results:**

The study showed significant increase in the prevalence of MAFLD with high thyroid stimulating hormone (TSH) levels (*P* < 0.01) and abnormal high-sensitive C-reactive protein (hsCRP) levels (*P* < 0.01). The proportion of MAFLD patients decreased significantly with the rise of free thyroxine (FT4) (*P* = 0.04), thyrotropin receptor antibodies (TRAb) (*P* < 0.01), anti-thyroglobulin antibodies (TgAb) (*P* < 0.01), and thyroid peroxidase antibodies (TPOAb) levels (*P* < 0.01). Based on logistic regression analysis, MAFLD was significantly associated with lower levels of TgAb (*P* < 0.01), TPOAb (*P* < 0.01), and higher levels of hsCRP (*P* < 0.01) in male. In female, elevated TgAb (*P* < 0.01) may be a protective factor, while higher levels of hsCRP (*P* < 0.01) showed increased risk of MAFLD. Logistic models were adjusted for age, BMI, SBP, DBP, FBG, ALT, AST, TC, TG, LDL, HDL.

**Conclusions:**

Taken together, TgAb may be a potential protective factor for MAFLD and elevated hsCRP level should be considered as an independent risk factor for MAFLD in both genders. TPOAb also demonstrated protective effect, but only in male. The prevalence of MAFLD increased with higher TSH levels and lower FT4, TRAb levels, but no significant association were found. However, Our findings provide a new insight into the pathogenesis of MAFLD by further investigating the impact of THs, thyroid autoimmunity, and inflammation on MAFLD patients.

## Introduction

Recently, nonalcoholic fatty liver disease (NAFLD) has been renamed as metabolic dysfunction associated fatty liver disease (MAFLD), underlining the association of fatty liver disease with metabolic dysfunction (1, 2). MAFLD is a worldwide epidemic with a prevalence around 25% in overall population, poses enormous health burden globally (3). The pathogenic process of MAFLD is believed to originate from a potential systemic metabolic dysfunction state. MAFLD is significantly related to metabolic abnormalities (4), including type 2 diabetes mellitus (T2DM) (5) and cardiovascular disease (CVD) (6). Therefore, identifying the risk factors of MAFLD is necessary for the development of new screening methods, prevention or treatment strategies.

Thyroid hormones (THs) play a fundamental role in metabolism. Thyroid dysfunction is considered to be associated with type 1 diabetes mellitus (T1DM), T2DM (7), and metabolic syndrome (MetS) (8). THs targets the liver to regulate body weight, lipogenesis, lipid metabolism, and insulin resistance (IR). Many liver diseases, such as liver fibrosis and cirrhosis hepatocellular carcinoma (9) are affected by THs. Recently, the association between MAFLD and THs has been discussed and is considered controversial. Some studies have shown that the prevalence of MAFLD is positively correlated with thyroid stimulating hormone (TSH), and TSH level may be an important risk factor for MAFLD (10). Nevertheless, other studies have suggested that the association between MAFLD and TSH is mediated by the characteristics of MetS, thus TSH levels cannot be used as independent risk factors for NAFLD (11). Other THs, including free triiodothyronine (FT3), free thyroxine (FT4), and FT3/FT4 ratio have proven to be associated with MAFLD (12, 13). However, this association of MAFLD with FT3 and FT4 levels are heterogeneous among population (10, 11).

Immune and inflammatory pathways also have crucial roles in the pathogenesis of MAFLD. Both innate immunity (14) and adaptive immunity (15) contribute to the progression of MAFLD. In autoimmune thyroid diseases (AITD), thyroid autoantibodies not only cause damage to thyroid tissues, but may also have potential out-thyroid actions (16). Recently, studies have shown that the presence of thyroid antibodies are involved in glucose and lipid metabolic disorders (17, 18). In addition, thyroid autoimmune may have a tumor-promoting role in breast cancer carcinogenesis (19) and may act as an independent CVD risk factor by promoting chronic inflammation (20). The relationship between thyroid antibodies and MAFLD remains unclear and few previous studies have been done. Moreover, metabolic inflammation is one of the commonly reported underlying mechanisms of MAFLD (21, 22). Several inflammatory biomarkers including high-sensitive C-reactive protein (hsCRP) are known to be associated with MAFLD (23). Although hsCRP has been used clinically as an inflammatory marker in the diagnosis of MAFLD, their relationship in the Chinese population has not been well studied.

Thus, our study aims to further clarify the association between THs, thyroid autoantibodies and MAFLD. Moreover, we attempt to provide evidence to support hsCRP as an independent predictor of MAFLD among a wider population.

## Materials and Methods

### Study Population and Design

**Figure 1:**
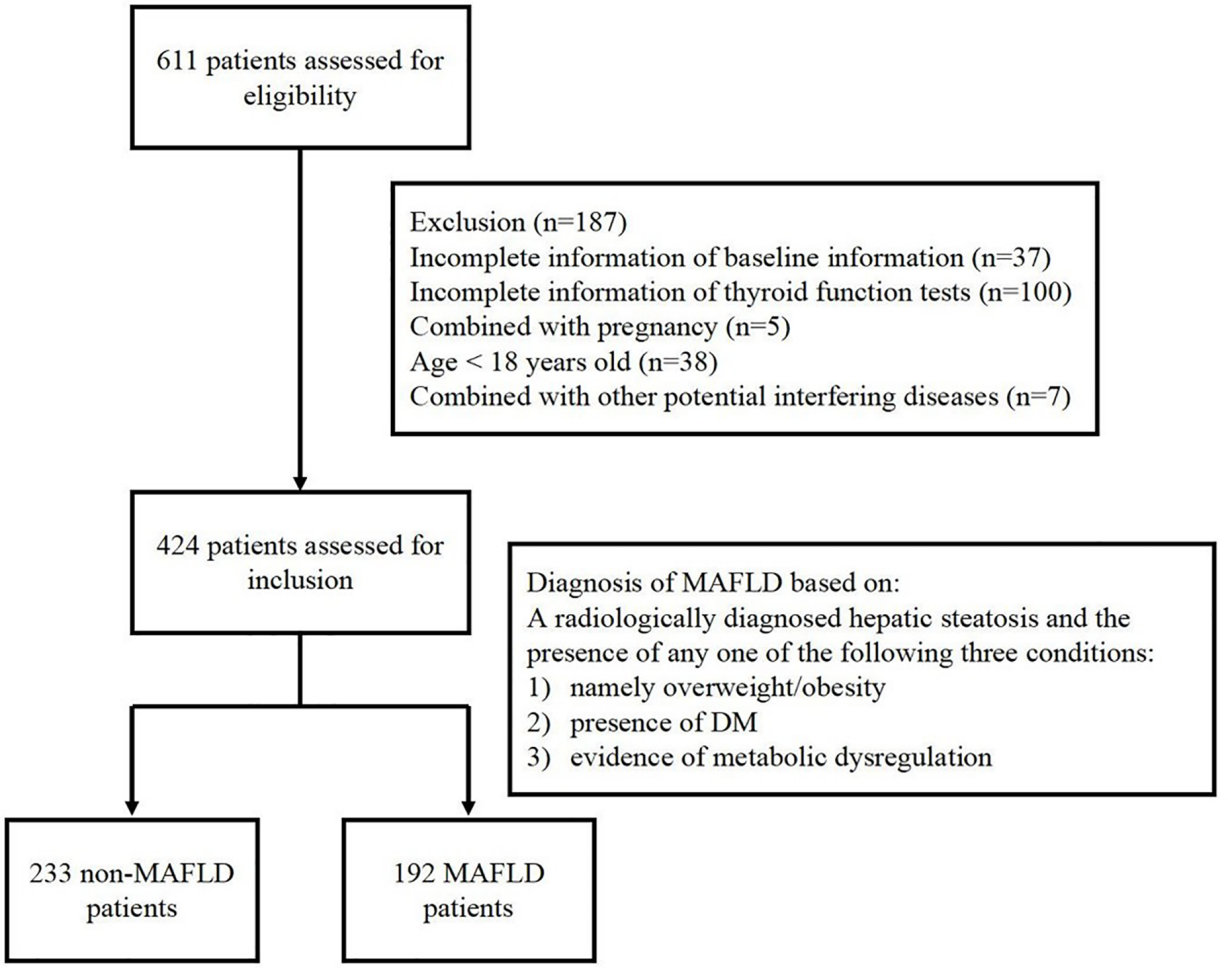
Flowchart of the inclusion and exclusion of participants in this study.

This study retrospectively collected data from 424 inpatients at Department of Endocrinology, Soochow University Affiliated No.1 People’s Hospital, from August 2019 to November 2019. Among the selected patients, 356 patients were diagnosed with T2DM, 9 were diagnosed T1DM, 2 were diagnosed with MetS, 8 were diagnosed with obesity, 20 were diagnosed with hyperthyroidism and hypertension, dyslipidemia, facial neuritis patients are involved in the rest subjects. There are 7 patients being excluded because of their potential interfering disease (24), including 3 patients with adrenal adenoma, 2 with polycystic ovary syndrome, 1 with acromegaly and 1 with postoperative pituitary tumor. The selected participants include euthyroid patients, hyperthyroid patients and hypothyroid patients. This study was approved by the Institutional Ethics Committee of Soochow University Affiliated No.1 People’s Hospital and conformed to the Declaration of Helsinki. Written informed consent was obtained from all patients. Selected patients were divided into MAFLD group and non-MAFLD group according to the novel consensus of diagnostic criteria in 2020. The inclusion and exclusion of participants in this study is shown in **Figure 1**.

### Clinical and Laboratory Assessments

Demographic information of inpatients was collected on the first day of residency, consisting of ID, diagnosis, admission date, sex, age (years), weight (kg), height (m), body mass index (BMI) (kg/m^2^), systolic blood pressure (SBP) (mmHg), diastolic blood pressure (DBP) (mmHg). A thyroid function test of thyroid hormone and antibody levels, TSH (ulU/ml), FT4 (ng/dL), FT3 (pg/ml), TRAb (u/L), TgAb (IU/ml), thyroid peroxidase antibodies (TPOAb) (IU/ml) were measured by an enzyme-linked immunosorbent assay kit (IC company, America). Other biochemical indicators, hsCRP (mg/L), platelet count (PC) (×10^9^/L), fasting blood glucose (FBG) (mmol/L), serum alanine aminotransferase (ALT) (U/L), aspartate aminotransferase (AST) (U/L), total cholesterol (TC) (mmol/L), triglyceride (TG) (mmol/L), low-density lipoprotein (LDL) (mmol/L), high density lipoprotein (HDL) (mmol/L) were estimated in a biochemistry analysis with Hitachi 7600 automatic biochemical analyzer.

Normal reference ranges for TSH, FT4, FT3, TRAb, TgAb, TPOAb, hsCRP and PC were 0.38–5.57 ulU/ml, 0.78–1.86 ng/dL, 1.8–3.8 pg/ml, 0.1–1.5 IU/ml, 0–14.58 IU/ml, 0–2.6 IU/ml, 0–3.0 mg/L, and (120–350) × 10^9^/L, respectively.

### Definitions

Patients were diagnosed as MAFLD based on a radiologically diagnosed hepatic steatosis and the presence of any one of the following three conditions: namely overweight/obesity (BMI ≥23 kg/m^2^ in Asian populations), presence of diabetes mellitus (DM), or evidence of metabolic dysregulation (1). The metabolic dysregulation was defined as the presence of two or more following conditions: 1) Waist circumference ≥102 cm in men or 88 cm in women; 2) Blood pressure ≥130/85 mmHg or specific drug treatment; 3) TG ≥1.70 mmol/L or specific drug treatment; 4) High density lipoprotein cholesterol <1.0 mmol/L for male or <1.3 mmol/L for female; 5) Prediabetes (i.e., fasting glucose levels 5.6 to 6.9 mmol/L, or 2-h post-load glucose levels 7.8 to 11.0 mmol/L or HbA1c 5.7% to 6.4%; 6) Homeostasis model assessment-insulin resistance score ≥2.5; 7) C-reactive protein level >2 mg/L. The non-MAFLD population referred to patients who do not meet any of the above conditions. All included patients had undergone an abdominal ultrasonography scan during hospitalization to detect the presence of hepatic steatosis. With Philip IU 22 ultrasound machine, hepatic ultrasonography was conducted by trained ultrasonic diagnostic specialists who were unaware of the objective of this study.

### Statistical Analysis

Medians and quartiles were used for continuous variables that do not follow normal distribution, and they were compared using Wilcoxon Signed Rank Test. Categorical variables were presented as absolute numbers and proportions, and they were compared using chi-square test. Generalized linear models were used to explore the linear relationship between serum THs, thyroid antibodies, inflammatory biomarkers and prevalence of MAFLD.

The associations between TSH, FT4, FT3, TPOAb, TRAb, TgAb, hsCRP, PC, and presence of MAFLD were analyzed using logistic regression models. Model 1 adjusted sex, age and BMI. Model 2 adjusted the factors included by model 1, together with SBP, FBG, TC, TG, ALT, AST, LDL, and HDL. A significant level of 0.05 was used. All statistical analyses were performed using SAS version 9.4 (SAS Institute).

## Results

### Baseline Characteristics of the Patients in the Metabolic Dysfunction-Associated Fatty Liver Disease Group and Non-Metabolic Dysfunction-Associated Fatty Liver Disease Group

A total of 424 participants at Department of Endocrinology, the First Affiliated Hospital of Soochow University from August 2019 to November 2019 participated in this study. There were 233 cases in the non-MAFLD group and 192 cases in the combined MAFLD group. The baseline information of the non-MAFLD group and the MAFLD group were compared in **Table 1**.

**Table 1:** Baseline information of the non-metabolic dysfunction associated fatty liver disease (MAFLD) group and MAFLD group [median (interquartile range)].

Characteristics	non-MAFLD (*n* = 233)	MAFLD (*n* = 192)	*P-*value
Sex (Male)	132	109	0.98
Age (years)	58.0 (47.0–67.0)	52.5 (36.5–65.0)	<0.01*
BMI (kg/m^2^)	23.4 (20.8–25.3)	26.1 (24.0–29.1)	<0.01*
SBP (mmHg)	125.0 (115.0–137.0)	126.0 (120.0–136.0)	0.28
DBP (mmHg)	78.0 (72.0–85.0)	81.0 (75.0–89.0)	<0.01*
FBG (mmol/L)	6.1 (5.2–8.3)	8.1 (6.4–10.6)	<0.01*
ALT (U/L)	18.2 (13.0–28.7)	25.0 (16.7–44.0)	<0.01*
AST (U/L)	17.2 (13.8–24.8)	20.5 (15.1–29.5)	<0.01*
TC (mmol/L)	4.3 (3.5–5.0)	4.6 (3.9–5.4)	<0.01*
TG (mmol/L)	1.1 (0.8–1.7)	2.0 (1.4–3.0)	<0.01*
LDL (mmol/L)	2.5 (1.9–3.2)	2.8 (2.1–3.5)	0.01*
HDL (mmol/L)	1.0 (0.9–1.3)	0.9 (0.8–1.0)	<0.01*
PC (× 10^9^/L)	196.0 (164.0–232.0)	198.5 (167.5–234.5)	0.43

BMI, body mass index; SBP, systolic blood pressure; DBP, diastolic blood pressure; FBG, fasting blood glucose; ALT, alanine aminotransferase; AST, aspartate aminotransferase; TC, total cholesterol; TG, triglyceride; LDL, low-density lipoprotein; HDL, high-density lipoprotein, PC, platelet count.

*Statistically significant values with P < 0.05.

Study participants with MAFLD tend to be younger, with lower levels of HDL and higher levels of BMI, DBP, FBG, ALT, AST, TC, TG, and LDL in comparison with the non-MAFLD group, and the differences were statistically significant (*P* < 0.01). No obvious difference was found between the two groups in terms of gender, SBP and PC (**Table 1**).

### Comparison of Metabolic Dysfunction-Associated Fatty Liver Disease Prevalence under Different Thyroid Hormones, Thyroid Autoantibodies, and Inflammatory Biomarkers Levels

**Figure 2:**
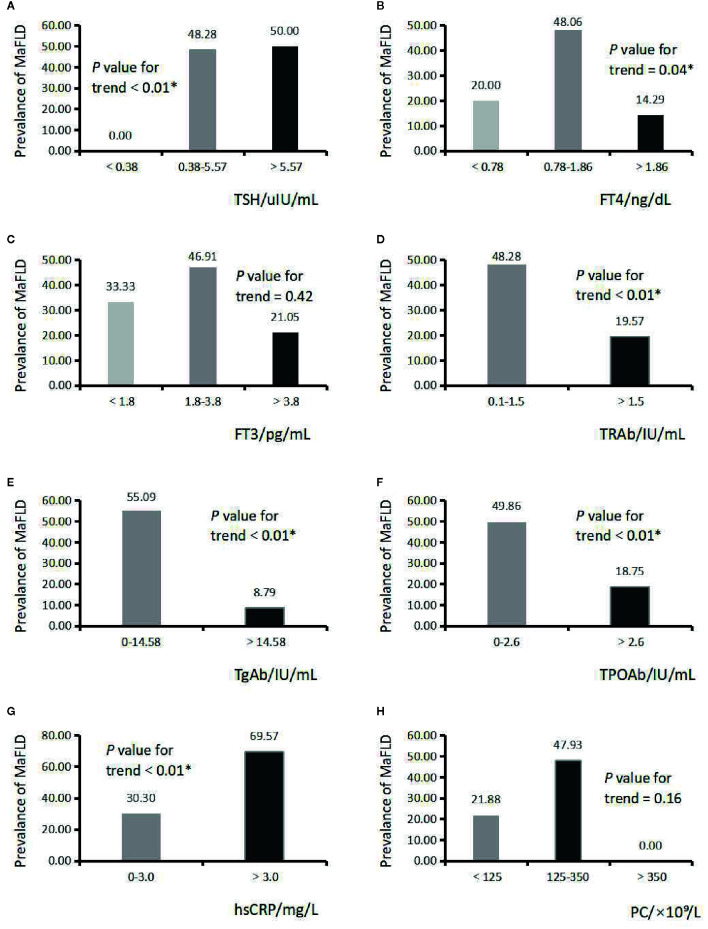
Serum thyroid hormones, thyroid autoantibodies, inflammatory biomarkers, and the proportions of metabolic dysfunction associated fatty liver disease (MAFLD) patients. **(A)** Serum thyroid stimulating hormone (TSH) level and the proportion of MAFLD patients. *P* trend < 0.01*; **(B)** Serum FT4 level and the proportion of MAFLD patients. *P* trend = 0.04*; **(C)** Serum FT3 level and the proportion of MAFLD patients. *P* trend = 0.42; **(D)** Serum TRAb level and the proportion of MAFLD patients. *P* trend < 0.01*; **(E)** Serum TgAb level and the proportion of MAFLD patients. *P* trend < 0.01*; **(F)** Serum TPOAb level and the proportion of MAFLD patients. *P* trend < 0.01*; **(G)** Serum hsCRP level and the proportion of MAFLD patients. *P* trend < 0.01*; **(H)** PC and the proportion of MAFLD patients. *P* trend = 0.16.

The associations between the levels of THs, thyroid autoantibodies, inflammatory biomarkers and the proportions of MAFLD patients were investigated. As is shown in **Figure 2**, there was a significant increase in the prevalence of MAFLD with the rise of serum TSH (0.00, 48.28, 50.00%; *P* for trend < 0.01) and hsCRP (30.30, 69.57%; *P* for trend < 0.01) level. Notably, the proportion of MAFLD patients decreased significantly with the rise of serum FT4 (20.00, 48.06, 14.29%; *P* for trend 0.04), TRAb (48.28, 19.57%; *P* for trend < 0.01), TgAb (55.09, 8.79%; *P* for trend < 0.01) and TPOAb (49.86, 18.75%; *P* for trend < 0.01) level.

### Logistic Regression Analysis of the Association Between Metabolic Dysfunction-Associated Fatty Liver Disease and Metabolic Variables

Logistic regression analysis was applied to further delineate the relationship between MAFLD and metabolic variables (**Table 2**). In age and BMI-adjusted logistic models, the ORs of combining MAFLD were significantly lower among participants with higher serum levels of TgAb, TPOAb in male subjects. In female patients, the ORs of combining MAFLD were significantly lower among participants with higher serum levels of FT4, TRAb, TgAb, TPOAb. Compared with normal serum hsCRP level of participants, the OR for exceeding hsCRP level was significantly higher in both genders.

**Table 2:** Logistic regression analysis for the risks of metabolic dysfunction associated fatty liver disease (MAFLD).

Characteristics	Male with Model 1^a ^		Female with Model 1^a^	
	OR (95%CI)	*P*-value	OR (95%CI)	*P*-value
TSH (ulU/ml)	0.70 (0.14,3.57)	0.67	1.04 (0.28,3.95)	0.95
FT4 (ng/dL)	0.58 (0.10,3.26)	0.53	0.14 (0.03,0.71)	0.02*
FT3 (pg/ml)	1.83 (0.29,11.60)	0.52	0.44 (0.10,1.94)	0.28
TRAb (IU/ml)	0.41 (0.14,1.17)	0.09	0.11 (0.02,0.56)	0.01*
TgAb (IU/ml)	0.03 (0.01,0.13)	<0.01*	0.04 (0.01,0.13)	<0.01*
TPOAb (IU/ml)	0.09 (0.02,0.46)	<0.01*	0.29 (0.12,0.70)	0.01*
hsCRP (mg/L)	3.38 (1.77,6.46)	<0.01*	4.07 (2.03,8.18)	<0.01*
PC (× 10^9^/L)	0.69 (0.15,3.17)	0.99	0.29 (0.09,1.01)	0.05
**Characteristics**	**Male with Model 2^b ^**		**Female with Model 2^b^**	
	**OR (95%CI)**	***P-*value**	**OR (95%CI)**	***P-*value**
TSH (ulU/ml)	0.82 (0.13,5.13)	0.83	1.69 (0.35,8.25)	0.52
FT4 (ng/dL)	0.64 (0.10,4.30)	0.65	0.21 (0.04,1.31)	0.10
FT3 (pg/ml)	0.99 (0.11,8.55)	0.99	0.76 (0.13,4.50)	0.76
TRAb (IU/ml)	0.55 (0.17,1.75)	0.31	0.22 (0.04,1.15)	0.07
TgAb (IU/ml)	0.02 (0.003,0.11)	<0.01*	0.05 (0.01,0.19)	<0.01*
TPOAb (IU/ml)	0.05 (0.01,0.35)	<0.01*	0.63 (0.23,1.71)	0.36
hsCRP (mg/L)	3.03 (1.48,6.19)	<0.01*	3.92 (1.68,9.11)	<0.01*
PC (× 10^9^/L)	0.65 (0.11,3.86)	0.99	0.27 (0.05–1.37)	0.11

TSH, thyroid-stimulating hormone; FT4, free thyroxine; FT3, free triiodothyronine; TRAb, anti-TSH receptor antibody; TgAb, thyroglobulin antibodies; TPOAb, thyroid peroxidase antibodies; hsCRP, high-sensitive C-reactive protein; PC, platelet count; FBG, fasting blood glucose; TC, total cholesterol; TG, triglyceride; LDL, low-density lipoprotein; HDL, high-density lipoprotein.

*Statistically significant values with P < 0.05.

^a^A univariable logistic regression model adjusted for age and BMI.

^b^A multivariate logistic regression model adjusted for age, BMI, SBP, DBP, FBG, ALT, AST, TC, TG, LDL, HDL.

After further adjustment for SBP, DBP, FBG, ALT, AST, TC, TG, LDL, and HDL in model 2, it’s suggested that hsCRP might be an independent risk factor for MAFLD, while TgAb might be a protective factor, as evidenced by higher OR for hsCRP and lower OR for TgAb in both genders. Results obtained from analysis in male demonstrated protective effects of abnormal TgAb, TPOAb levels (OR, 95%CI; 0.02, 0.003–0.11 for TgAb; 0.05, 0.01–0.35 for TPOAb), while higher hsCRP levels maintained as risk factor (OR, 95%CI; 3.03, 1.48–6.19). In female, elevated TgAb was a protective factor (OR, 95%CI; 0.05, 0.01–0.19), while higher hsCRP levels maintained as risk factor (OR, 95%CI; 3.92, 1.68–9.11). Together, the present findings confirmed that TgAb may be a protective factor and hsCRP may be a risk factor in all study participants. Closer inspection of the table showed protective effects of TPOAb in male only.

### Baseline Characteristics of Subjects Stratified Respectively by Anti-Thyroglobulin Antibodies and High-Sensitive C-Reactive Protein Levels

**Table 3** shows the baseline characteristics of subjects stratified according to TgAb levels. As is shown in **Table 3**, number of patients combined with MAFLD were 184 (55.1%) and 9 (9.9%) for normal and abnormal TgAb levels respectively, which indicates participants with exceeding TgAb levels tend to be free from MAFLD. In addition, female patients had a higher possibility of abnormal TgAb levels. Furthermore, we explored that patients with elevated TgAb had lower DBP volume, lower FBG levels, lower TG levels and higher HDL levels compared to the normal TgAb group. In male, TgAb positive patients showed lower levels of FBG (*P* < 0.01) and higher HDL levels (*P* < 0.05). Female TgAb positive patients demonstrated lower levels of FBG (*P* < 0.01), TC (*P* = 0.02), TG (*P* < 0.01) and LDL (*P* < 0.05). We next set out to discover the thyroid status in TgAb positive subjects, among which 62 (68.89%) were euthyroidism, 17 (18.89%) were hyperthyroidism and 11 (12.22%) were hypothyroidism.

**Table 3:** Baseline characteristics of subjects stratified according to TgAb levels.

Characteristics^a^	Normal (*n* = 334)	Abnormal (*n* = 91)	*P-*value
Number of patients	184 (55.1)	9 (9.9)	<0.01*
Sex (Male)	205 (61.4)	36 (39.6)	<0.01*
Age (Years)	56.0(40.0–66.0)	56.0(43.0–64.0)	0.87
BMI (kg/m^2^)	24.5(22.5–27.0)	24.2(21.8–27.0)	0.25
SBP (mmHg)	126.0(118.0–137.0)	125.0(116.0–133.0)	0.56
DBP (mmHg)	80.0(74.0–87.0)	76.0(72.0–85.0)	0.02*
FBG (mmol/L)	7.3(5.8–9.9)	5.8(5.0–7.1)	<0.01*
ALT (U/L)	20.5(14.4–35.0)	20.3(14.7–38.3)	0.66
AST (U/L)	18.1(14.5–26.4)	18.0(14.2–28.0)	0.95
TC (mmol/L)	4.4(3.7–5.3)	4.2(3.6–5.0)	0.08
TG (mmol/L)	1.6(1.0–2.4)	1.2(0.9–1.9)	<0.01*
LDL (mmol/L)	2.8(2.1–3.3)	2.5(1.8–3.4)	0.14
HDL (mmol/L)	0.9(0.8–1.1)	1.0(0.9–1.2)	0.01*
PC (× 10^9^/L)	195.0(165.0–229.0)	206.0(168.0–252.0)	0.29
**Male**			
**Characteristics^a^**	**Normal** **(*n* = 205)**	**Abnormal** **(*n* = 36)**	***P-*value**
Number of patients	107 (52.2)	2 (5.6)	<0.01*
Age (Years)	52.0(39.0–64.0)	52.0(39.5–62.0)	0.87
BMI (kg/m^2^)	24.8(22.6–27.4)	25.0(22.7–27.0)	0.73
SBP (mmHg)	126.0(118.0–138.0)	125.0(118.0–132.5)	0.33
DBP (mmHg)	81.0(76.0–88.0)	76.0(71.0–88.5)	0.12
FBG (mmol/L)	7.5(5.9–9.8)	5.6(4.5–6.4)	<0.01*
ALT (U/L)	20.5(15.0–35.9)	21.1(16.6–31.6)	0.61
AST (U/L)	18.1(13.9–25.5)	18.1(13.0–27.8)	0.79
TC (mmol/L)	4.3(3.6–5.2)	4.3(3.5–5.0)	0.60
TG (mmol/L)	1.5(1.0–2.4)	1.5(1.0–2.3)	0.78
LDL (mmol/L)	2.7(2.0–3.3)	2.5(1.7–3.7)	0.80
HDL (mmol/L)	0.9(0.8–1.1)	1.0(0.9–1.3)	0.05*
PC (× 10^9^/L)	192.0(164.0–220.0)	213.5(162.0–251.5)	0.25
**Female**			
**Characteristics^a^**	**Normal** **(*n* = 129)**	**Abnormal** **(*n* = 55)**	***P-*value**
Number of patients	77 (59.7)	6 (10.9)	<0.01*
Age (Years)	61.0(49.0–70.0)	57.0(47.0–69.0)	0.29
BMI (kg/m^2^)	24.2(22.2–26.6)	23.5(20.8–26.9)	0.41
SBP (mmHg)	125.0(117.0–135.0)	125.0(115.0–135.0)	0.74
DBP (mmHg)	79.0(72.0–86.0)	78.0(72.0–82.0)	0.27
FBG (mmol/L)	7.2(5.5–9.9)	5.9(5.2–7.4)	<0.01*
ALT (U/L)	20.6(14.1–33.2)	20.3(13.5–41.8)	0.83
AST (U/L)	18.0(15.1–27.0)	18.0(14.5–28.7)	0.82
TC (mmol/L)	4.7(3.9–5.3)	4.2(3.6–4.9)	0.02*
TG (mmol/L)	1.7(1.2–2.4)	1.1(0.8–1.6)	<0.01*
LDL (mmol/L)	2.9(2.2–3.3)	2.4(1.8–3.4)	0.05*
HDL (mmol/L)	1.0(0.9–1.2)	1.1(0.8–1.2)	0.52
PC (× 10^9^/L)	205.0(167.0–250.0)	201.0(172.0–252.0)	0.97

BMI, body mass index; SBP, systolic blood pressure; DBP, diastolic blood pressure; FBG, fasting blood glucose; ALT, alanine aminotransferase; AST, aspartate aminotransferase; TC, total cholesterol; TG, triglyceride; LDL, low-density lipoprotein; HDL, high-density lipoprotein; PC, platelet count.

*Statistically significant values with P < 0.05.

^a^Variables are expressed as median (interquartile range).

Sectionalized according to hsCRP levels, the prevalence of MAFLD was significantly higher among people with higher hsCRP levels. Also, we have noticed that participants with exceeding hsCRP levels had lower HDL levels and higher BMI, FBG, ALT, AST, TG, LDL, and PC levels (**Table 4**).

**Table 4:** Baseline characteristics of subjects stratified according to hsCRP levels.

Characteristics^a^	Normal(*n*=264)	Abnormal(*n*=161)	*P-* value
Number of patients	80 (30.3)	112 (69.6)	<0.01*
Sex (Male)	161 (61.0)	80 (49.7)	0.02
Age (Year)	56.0(43.5–65.5)	55.0(37.0–66.0)	0.26
BMI (kg/m2)	23.8(21.5–26.0)	25.9(23.7–29.2)	<0.01*
SBP (mmHg)	126.0(117.5–138.0)	126.0(118.0–135.0)	0.56
DBP (mmHg)	79.0(74.0–87.0)	80.0(73.0–87.0)	0.91
FBG (mmol/L)	6.4(5.3–9.2)	7.3(6.0–9.8)	<0.01*
ALT (U/L)	20.3(14.5–30.2)	22.6(14.8–44.0)	0.05*
AST (U/L)	17.8(14.2–25.1)	19.5(14.7–29.8)	0.04*
TC (mmol/L)	4.3(3.5–5.0)	4.6(3.7–5.4)	0.04*
TG (mmol/L)	1.3(0.9–2.1)	1.8(1.2–2.8)	<0.01*
LDL (mmol/L)	2.6(1.9–3.2)	2.9(2.2–3.6)	<0.01*
HDL (mmol/L)	1.0(0.8–1.2)	0.9(0.8–1.1)	<0.01*
PC (× 10^9^/L)	191.0(163.0–227.0)	207.0(172.0–251.0)	0.01*

BMI, body mass index; SBP, systolic blood pressure; DBP, diastolic blood pressure; FBG, fasting blood glucose; ALT, alanine aminotransferase; AST, aspartate aminotransferase; TC, total cholesterol; TG, triglyceride; LDL, low-density lipoprotein; HDL, high-density lipoprotein; PC, platelet count.

*Statistically significant values with P < 0.05.

^a^Variables are expressed as median (interquartile range).

## Discussion

The present study demonstrated an independently negative association between abnormal TgAb level and MAFLD in all population, indicating TgAb may be a protective factor for MAFLD. Our results also confirmed a strong association between hsCRP elevation and MAFLD, independent from cardiometabolic risk factors. Despite the fact that differences on the percentage of serum TSH, FT4 and TRAb levels were found between the non-MAFLD group and the MAFLD group, TSH, FT4, and TRAb were not independent risk factors for MAFLD according to the results of logistic regression. Furthermore, differences on age, BMI, DBP, FBG, ALT, AST, TC, TG, LDL, and HDL were shown among the two groups. TPOAb demonstrated reduced risks for MAFLD in male but not in female. We have noticed that female patients had significantly higher TgAb abnormalities, while DBP, FBG, TG was significantly lower in patients with abnormal TgAb levels. Elevated HDL was also found in TgAb positive patients. Further stratification by sex demonstrated lower FBG and higher HDL levels in male, while TgAb positive female patients showed lower FBG, TC, TG, and LDL levels. Importantly, sex, age, BMI, FBG, ALT, AST, TG, LDL, HDL, and PC differed along with changes in hsCRP levels.

Thyroid function plays a significant role in the regulation of energy and lipid metabolism. MetS, body weight, IR and hyperlipidemia are proven to show a correlation with thyroid function (25, 26). Moreover, THs have direct influences on hepatic lipid, cholesterol, and glucose metabolism (27), consisting with the underling mechanism of MAFLD. Thus, we have evidenced that thyroid function may have a role in the pathogenesis of MAFLD. Recently, many studies have investigated the association between THs and MAFLD while they showed incongruous results. Several studies discovered elevated TSH levels were significantly associated with an increased incidence of MAFLD (28, 29) while some contradicted (30, 31). What’s more, results in a large longitudinal cohort study showed that the correlation of TSH with MAFLD was not independent of cardiometabolic confounders (32). Another study involving 10,539 individuals suggested that the association between TSH and MAFLD was mediated by the characteristics of MetS (waist circumference, HDL levels, triglycerides levels, DM, and systemic arterial hypertension) (33). Notably, an inverse correlation between FT4 level and risk were found in some studies (12, 29, 34). By contrast, no significant association between them were found in other studies (35, 36). The correlation between FT3 and MAFLD also remains disputable. Some studies reported a positive correlation between FT3 level and risk of MAFLD (35, 37) while some studies did not find any significant association between them (29, 36, 38). Our results confirmed a significant increase in MAFLD rate with increased serum TSH. We next characterized that TSH was not an independent risk factor of MAFLD. In order to examine the association between FT3, FT4, and MAFLD, we made a comparison between the MAFLD and non-MAFLD group. These data suggest no association of neither FT3 nor FT4 with MAFLD after adjustment for cardiometabolic confounders. Overall, our research demonstrated no significant difference in THs levels between participants with and without MAFLD, consisting with the recent systematic review and meta-analysis (36). Several underlining reasons may explain the discrepancy between previous studies. To begin with, the regions and races of the population in these studies were different. The researches by Chung (28), Bano (34), and Van den Berg (12) were performed in Europe while the remaining studies were performed in China. Moreover, the characteristics of study population were different among studies. Chung (28), Lee (32) focused on hypothyroid groups while others studied on euthyroid subjects. In addition, variations in the definition of hypothyroidism and the measurement of THs existed (36). Our studies serve as a proof-of-concept data that THs may not be independent risk factors of MAFLD.

Abnormal serum thyroid autoantibody levels are not only frequently found in patients with AITD but also in subjects without manifesting thyroid dysfunction (19). Previous studies on the Chinese general population have reported that the positive rates of serum TgAb and TPOAb were 12.6% and 11.5%, respectively (39). Moreover, the prevalence of thyroid autoantibodies increases with age. A study on East China population with a mean age of 60 discovered that the prevalence of TPOAb and/or TgAb was 17.4% (40). Another study showed that among adults more than 65 years old, 19.30% were positive for at least one thyroid autoantibody (41). Genetic determinants influenced by iodine intake, stress, chemical contaminants, and infectious organisms may cause heterogeneity in susceptibility to autoimmune thyroid disease. A previous study in Xinjiang province, China reported a prevalence of TPOAb and/or TgAb positive of 32.1% (21.2% in men and 37% in women) (42). Our subjects originated from Suzhou, Jiangsu province, China. National monitoring data showed that all provinces and 95% of counties in China have eliminated iodine deficiency diseases in 2010. Based on the results of the 2011 National Iodine Deficiency Disease Survey, pregnant women in Jiangsu have optimal iodine intakes (150–249 μg/L) while school age children have excessive iodine intakes (≥300 μg/L) (43). Recent study on Chinese population showed that excessive iodine intake has an inverse relationship with TPOAb positivity and is associated with a higher prevalence of positive TgAb (44). Excessive iodine intake may contribute to the relatively high positive rate of TgAb (21.4% in general population, 16.8% in men and 30.0% in women) in our study.

Thyroid autoantibodies were believed to have potential systemic functions (16). We observed significant decrease in MAFLD prevalence with the rise of serum TRAb and TgAb. We further discovered that TgAb may be a protective factor for MAFLD in both genders. To our notice, there is only one study by Chen (45) focusing on the association between thyroid autoantibodies and NAFLD so far. In contrast, the study revealed a positively association between AITD and the prevalence of NAFLD. The discordance of results may be attributed to different study population and diagnosis criteria. Chen’s study involved euthyroid patients with normal TSH levels only, while we included patients with different thyroid status. Moreover, AITD was defined as serum TPOAb and/or TgAb positivity, and TPOAb and/or TgAb positivity together with characteristic US features in Chen’s study. However, we analyzed the association of thyroid autoantibodies with MAFLD respectively. In addition, Chen’s study applied diagnosis criteria of NAFLD, while we defined MAFLD according to the novel consensus of diagnostic criteria in 2020. A recent study has noted difference between the two diagnostic criteria in real world (46). We tentatively put forward that the potential mechanism may be related to glucose and lipid metabolism. Importantly, we identified that patients with elevated TgAb levels had significantly lower FBG in both genders. Our results consist with a recent study based on Chinese nation survey which showed serum TgAb positivity may imply a reduced risk of impaired FBG in euthyroid men and a reduced risk of hypertriglyceridemia in euthyroid women (17). Another study on Portuguese population discovered a negative association of TPOAb positivity with MetS and its TG component. In contrast, no association of TgAb was not found with MetS or its components (20). Therefore, not only is there a lack of consistent findings in the relationship between thyroid autoantibody positivity and the prevalence of MAFLD, but also a lack of understanding of the respective roles of TPOAb and TgAb, which requires further study. It remains unclear to which degree MAFLD attributed to TgAb. Here, we tentatively bring forward two possible mechanisms. To begin with, a recent study found that carbonic anhydrase (CA) is genetically related to TgAb (47). Decreased CA activity may induce the production of TgAb *via* high levels of iodine uptake (48). CA is the main diver of hepatic gluconeogenesis and *de novo* lipogenesis by providing HCO3- for the first reaction (49, 50). Studies have identified the role of CA in obesity and NAFLD, suggesting CA inhibitors as potential therapeutic targets (51, 52). Therefore, the protective effect of serum TgAb positive expression on the occurrence of MAFLD may be due to potentially abnormal CA activity. Secondly, studies on experimental autoimmune thyroiditis (EAT) mouse discovered up-regulated levels of interferon gamma (IFN-γ), and found that the production of TgAb was related to IFN-γ (53, 54). Reduced production IFN-γ may lead to elevated levels of FBG and TG in high-fat diet mouse (55). Moreover, IFN-γ was considered to be an anti-inflammatory and antifibrotic cytokine, and had protective effect in MAFLD (56, 57). Thus, we speculate that IFN-γ also contribute to the protective effect of TgAb on MAFLD. Previous studies on Chinese male showed TPOAb positivity associated with higher ratio of estradiol to testosterone (58). The potential hepatoprotection effect of estradiol was confirmed in several studies. Clinical trials on diabetes patients also showed protective effects of estrogen therapy on MAFLD. The influence of estradiol on MAFLD may be attributed to its role in reducing hepatic fat accumulation as well as suppressing liver inflammation and fibrosis (59–61). It is possible that the protective role of TPOAb on MAFLD in male patients stem from higher estradiol levels.

Inflammation is commonly reported as one of MAFLD’s underlying mechanisms. Low-grade systemic inflammation is associated with elevated levels of various inflammatory markers including hsCRP (62). Several studies evaluating the level of hsCRP in MAFLD have shown the association between hsCRP and MAFLD after adjustment with cardiometabolic risk factors (63, 64). Moreover, a variety of features of MetS were associated with a systemic inflammatory response. Association between hsCRP and features of MetS, including TC, TG, BMI and glucose, were proven to exist in several studies (65, 66). Studies also found elevations of liver enzymes associated with higher hsCRP concentrations. This association raised the possibility that inflammatory processes accompanying MAFLD contribute to the systemic inflammation observed in subjects with MetS (67). Our findings confirm that patients with abnormal hsCRP have significantly increased BMI, FBG, ALT, AST, TG levels, and decreased HDL levels. These results verify hsCRP as an independent risk factor of MAFLD and its potential mechanism.

Our investigation further explored the association between thyroid function and MAFLD under a wider scale by including euthyroid, hyperthyroid, and hypothyroid patients. We evaluated the association between THs, thyroid autoantibodies, inflammatory biomarkers, and MAFLD. To our notice, few studies on the link of MAFLD including thyroid autoantibodies were done. Most of the previous studies focused on AITD but not TgAb or TPOAb elevation respectively. In attempt to minimize confounding factors, the statistical analyses were performed after adjustment for cardiometabolic risk factors (age, BMI, SBP, DBP, FBG, ALT, AST, TC, TG, LDL, HDL), which are widely-held MAFLD-determining factors. We also conducted analysis to evaluate the association between TgAb and hsCRP with well-known risk factors for MAFLD. These analyses contributed to further analysis of underlying mechanisms behind the findings. Undeniably, our study has some limitations. Subjects were sampled from only one hospital, implicating the demonstrated characteristics might not represent the entire Chinese population. Also, we diagnosed MAFLD patients according to the novel consensus of diagnostic criteria in 2020, but the utility of MAFLD diagnosis has not been tested and validated in real world.

## Conclusion

Overall, our studies establish that TgAb may be a potential protective factor for MAFLD and elevated hsCRP level should be considered as an independent risk factor for MAFLD in both genders. TPOAb also demonstrated protective effect, but only in male. The association may be attributed to glucose, lipid metabolism and inflammation. The prevalence of MAFLD increased with higher TSH levels and lower FT4, TRAb levels, but the association were not of statistical significance. Future iterations of the correlation between MAFLD and THs, together with thyroid autoantibodies may demonstrate greater potency.

## Data Availability Statement

The raw data supporting the conclusions of this article will be made available by the authors, without undue reservation.

## Ethics Statement

This study was approved by the Institutional Ethics Committee of Soochow University Affiliated No.1 People’s Hospital and conformed to the Declaration of Helsinki. Written informed consent was obtained from all patients.

## Author Contributions

XZ, YC, LC, and YL contributed to the conception and design of the study. RL, YC, and YD contributed to the data curation. XZ wrote the first draft of the manuscript. XZ, RL, YC, and YD wrote sections of the manuscript. All authors contributed to the article and approved the submitted version.

## Funding

This study was supported by the Suzhou Science and Technology Planning Project (grant no. SS201823) and the Suzhou Science and Technology Planning Project (grant no. SYS2019051).

## Conflict of Interest

The authors declare that the research was conducted in the absence of any commercial or financial relationships that could be construed as a potential conflict of interest.

## Abbreviations

AITD, autoimmune thyroid diseases; ALT, alanine aminotransferase; AST, aspartate aminotransferase; BMI, body mass index; CA, carbonic anhydrase; CVD, cardiovascular disease; DBP, diastolic blood pressure; DM, diabetes mellitus; FBG, fasting blood glucose; FT3, free triiodothyronine; FT4, free thyroxine; HDL, high density lipoprotein; hsCRP, high-sensitive C-reactive protein; IR, insulin resistance; LDL, low-density lipoprotein; MAFLD, metabolic dysfunction associated fatty liver disease; MetS, metabolic syndrome; NAFLD, nonalcoholic fatty liver disease; OR, odds ratio; PC, platelet count; SBP, systolic blood pressure; T1DM, type 1 diabetes mellitus; T2DM, type 2 diabetes mellitus; TC, total cholesterol; TG, triglyceride; TgAb, anti-thyroglobulin antibodies; THs, thyroid hormones; TPOAb, thyroid peroxidase antibodies; TRAb, thyrotropin receptor antibodies; TSH, thyroid stimulating hormone.
